# Editorial: State-of-the-Art Technology and Applications in Crop Phenomics

**DOI:** 10.3389/fpls.2021.767324

**Published:** 2021-10-05

**Authors:** Wanneng Yang, John H. Doonan, Malcolm J. Hawkesford, Tony Pridmore, Ji Zhou

**Affiliations:** ^1^National Key Laboratory of Crop Genetic Improvement, National Center of Plant Gene Research, Huazhong Agricultural University, Wuhan, China; ^2^Shenzhen Branch, Guangdong Laboratory for Lingnan Modern Agriculture, Genome Analysis Laboratory of the Ministry of Agriculture, Agricultural Genomics Institute at Shenzhen, Chinese Academy of Agricultural Sciences, Shenzhen, China; ^3^The National Plant Phenomics Centre, Institute of Biological, Environmental and Rural Sciences, Aberystwyth University, Aberystwyth, United Kingdom; ^4^Rothamsted Research, Harpenden, United Kingdom; ^5^School of Computer Science, University of Nottingham, Nottingham, United Kingdom; ^6^Cambridge Crop Research, National Institute of Agricultural Botany, Cambridge, United Kingdom; ^7^Academy for Advanced Interdisciplinary Studies, Jiangsu Collaborative Innovation Center for Modern Crop Production Co-sponsored by Province and Ministry, Nanjing Agricultural University, Nanjing, China

**Keywords:** high-throughput phenotyping, AI-driven phenotypic analysis, UAV (unmanned aerial vehicle), hyperspectral, X-ray micro-CT

Together with the rise of whole-genome sequencing of many plant species, large-scale and high-throughput plant phenotyping (HTP), as well as associated phenotypic analysis, has become a bottleneck that needs to be urgently relieved (Yang et al., 2020). Plant genetics and crop breeding can be accelerated by recent rapid advances through diverse technologies, from sensors to feature extraction, combined with increasing systems integration and decreasing costs in software and hardware systems. The integration of artificial intelligence (AI) driven techniques (e.g., deep learning and machine learning), computer vision, and big-data analytics, and their optimization for the life sciences, has opened doors to new opportunities for a broad plant science research community to develop step change solutions to bridge the gap between traits of interest and genomic information for novel biological discoveries (Tardieu et al., 2017; Zhao et al., 2019). In particular, recent developments in multi-factorial phenotypic models can be dynamically generated from large biological datasets to characterize phenotypic features, including the prediction of genotypic reaction to complex environments as well as genotype-based phenotypic changes across multiple seasons (Großkinsky et al., 2015; Furbank et al., 2019). Such methodological and technical advances have empowered plant scientists to unravel the genetics of complex phenotypes at the levels of cell, organ, tissue, plant, and population (Fiorani and Schurr, 2013).

The importance of this research area has been repeatedly discussed by the research community over the past decade, focusing on either methodological development or applications to varied plant questions. Several Research Topics concerning plant phenotyping were hosted by a series of Frontiers Research Journals, for example, “Drought Phenotyping in Crops: From Theory to Practice” in 2012, “Phenomics” in 2016, “Plant Phenotyping and Phenomics for Plant Breeding” in the year 2017, “Advances in High-Throughput Plant Phenotyping by Multi-platform Remote Sensing Technologies” in 2017, “Phenotyping at Plant and Cell Levels: the Quest for Tolerant Crop Development” in 2018, “High-throughput Field Phenotyping to Advance Precision Agriculture and Enhance Genetic Gain” in 2019, “Phenotyping; from Plant, to Data, to Impact and Highlights of the International Plant Phenotyping Symposium—IPPS 2018” in 2019, and “High-Throughput Phenotyping for Crop Improvement and Breeding” in 2020.

In this Research Topic, we promote recent the latest methodological improvements in crop phenotyping. The 19 research papers range from deep learning, x-ray computed tomography, hyperspectral imaging, to 3D imaging, as well as demonstrating wide applications of phenotyping technologies and phenotypic analysis in post-harvest quality control, breeding, plant research, and genomic selection.

In recent years, deep learning has become a widely adopted toolkit for vision-based trait recognition, classification and object segmentation in plants. Combined with a vision system, a CNN-based model, MobileNet-V2 was adopted to detect defective oranges and could be applied for in-line citrus sorting (Chen et al.). Another CNN model, MaskRCNN, can be used to detect horticultural crops with different degrees of ripeness using images taken in both greenhouse and field for yield-related trait analysis (Afonso et al.). Utilizing a sensor-to-plant greenhouse phenotyping platform capturing top-view images of lettuce, U-Net was used to segment image-based objects and quantify 15 shoot geometry and color traits to derive shoot growth traits (Du et al.). An improved Faster-RCNN recognized and located strawberries much better than ResNet50 and VGG16 (Zhou et al.), but with a higher computational cost when dealing with high-resolution images. In contrast, a fast version of a state-of-the-art plant counting model, TasselNetV2+ was adopted in quantifying numbers of wheat ears, maize tassels, and sorghum heads at 30 frames per second (fps) with an image resolution of 1980 × 1080 (Lu and Cao), which may be employed in real-time plant counting on a portable device in future. The ability of algorithms to function accurately, under diverse conditions and in real time, is essential if they are going to be used to control robotic pickers and similar machinery.

Deep learning has performed well for classification and object detection tasks in research settings. However, image data annotation can be immensely time-consuming and often leads to new bottleneck of generating sufficient and high-quality training datasets, which also could cause overfitting of deep learning models. Interestingly, to address this bottleneck, the combination of a Wasserstein generative adversarial network and gradient penalty (WGAN-GP) and label smoothing regularization (LSR) improved the classification accuracy of plant disease by 24.4% under limited training data (Bi and Hu).

For 2D imagery, machine learning techniques could be applied to analyze different organs of a plant. For example, deep learning was used to determine the number and size of soybean root nodules, and investigate nodule development under different silicon nutrition conditions (Chung et al.). Similarly, an improved CNN model, DeepLabv3+, was used to segment roots from soil and calculated parameters such as root length, surface area, diameter, and root volume with high accuracy (r^2^ = 0.9449) (Shen et al.). Beyond 2D imaging, x-ray computed tomography (CT) could be applied in monitoring the dynamic growth of potted potato tubers from initiation until harvest (Van Harsselaar et al.).

Sensors mounted on mobile platforms could provide flexible and cost-effective phenotyping solutions in field scenarios. For example, unmanned aerial vehicles (UAVs) and remote sensing can phenotype large-scale crop populations and are not constrained by the static infrastructures typical of greenhouses. For example, UAV-acquired multispectral traits could predict water use efficiency or nitrogen use efficiency and their impact on grain yield in winter wheat (Yang et al.). Using a line scan hyperspectral imaging system of 235 wavelengths from the visible and NIR spectral range, three machine learning-based models, partial least squares-discriminant analysis (PLS), support vector machine (SVM), and multilayer perceptron (MLP) were used to classify four weeds and showed a range of overall accuracy of 70–100% (Li et al.). In addition, hyperspectral image preprocessing, which included segmentation, image correction, and image space-spectral dimensional denoising, improved the classification accuracy of health and infected wheat seeds.

Based on multiple side-view images acquired in an indoor high-throughput plant phenotyping platform (University of Nebraska-Lincoln 3D Plant Phenotyping Dataset, UNL-3DPPD), an algorithm for 3D voxel-grid reconstruction, 3DPhenoMV, was developed to obtain the 3D phenotypes of maize and cotton (Das Choudhury et al.). Similarly, the reconstructed 3D phenotypic traits such as leaf angle and leaf area were used to remotely monitor the drought response in grapevines (Briglia et al.).

While morphological and physiological traits can be assessed non-destructively across large-scale populations, and repeatedly during plant growth and development, many current technologies are still at their infant stages and require active development. For example, many physiological processes are normally monitored based on single leaves, but the Plantarray 3.0 platform has demonstrated the possibility of undertaking such studies on intact plants. This platform was used to dynamically monitor growth and water use of the quinoa under saline conditions, which showed that the high-resolution functional phenotyping could promote the dissection of complex traits of abiotic stress tolerance (Jaramillo Roman et al.).

To handle the massive amount of phenotyping data generated by various imaging sensors, effective image, and data analysis pipelines are urgently needed. Image analysis software, 3DPheno-Seed&Fruit, was developed to extract 3D seed and fruit traits (Liu et al.) from X-ray CT scans. To process seed images acquired by lower cost flatbed scanners or digital cameras, an open-source application, SeedExtractor, was developed to determine seed shape, size, and color with a high speed of 2 s per image (Zhu et al.). Combined with genome-wide association analysis (GWAS), this tool was successfully applied to identify known loci controlling rice seed length and width. To process the huge amounts of data generated by an outdoor high-throughput phenotyping platform, an analytical pipeline SpaTemHTP, composed of three modules, detection of outliers, imputation of missing values, and mixed-model genotype adjusted means computation with spatial adjustment, was developed to efficiently process the temporal phenotyping data for the further genetic analysis (Kar et al.).

Novel image-based feature or derived secondary traits have the power to decipher the complex genetic architecture of drought tolerance in maize (Wu et al., 2021) and can improve genome selection (GS) models. A good example is spectral reflectance data collected using a handheld multi-spectral radiometer: such secondary traits can improve the prediction accuracy by 20% for grain yield and 12% for grain protein content of spring wheat, which showed that combining HTP and genome selection in a plant breeding program could potentially improve the genetic gain by increasing the selection accuracy and reducing the breeding cycle time (Sandhu et al.).

This Research Topic highlights the latest methodological developments of phenotyping and phenotypic analysis in plant research, ranging from the cell to the population level. By using available analytic techniques such as machine learning/deep learning and computer vision, a systematic approach to carry out indoor and in-field phenotyping is formed, capable of answering biological questions through feature extraction, trait analysis, dynamic modeling. As a fast-moving area, plant phenotyping requires efforts from plant biologists, hardware and software engineers, bioinformaticians, and data analysts, contributing to both applied and basic plant sciences. Hence, we believe that the Research Topic not only presents the state-of-the-art technological advances, but also demonstrates a promising approach to utilize high-quality phenotypic information in plant research, enabling us to exploit available genomic resources to develop crop varieties with desired qualities in the context of global climate change.

## Author Contributions

WY initially drafted the manuscript with inputs from other Topic Editors. All the Topic Editors read and approved the final manuscript. All authors contributed to the article and approved the submitted version.

## Conflict of Interest

The authors declare that the research was conducted in the absence of any commercial or financial relationships that could be construed as a potential conflict of interest.

## Publisher's Note

All claims expressed in this article are solely those of the authors and do not necessarily represent those of their affiliated organizations, or those of the publisher, the editors and the reviewers. Any product that may be evaluated in this article, or claim that may be made by its manufacturer, is not guaranteed or endorsed by the publisher.
